# Post-bronchiolitis wheezing is associated with toll-like receptor 9 rs187084 gene polymorphism

**DOI:** 10.1038/srep31165

**Published:** 2016-08-08

**Authors:** Kirsi Nuolivirta, Sari Törmänen, Johanna Teräsjärvi, Juho Vuononvirta, Petri Koponen, Matti Korppi, Merja Helminen, Ville Peltola, Qiushui He

**Affiliations:** 1 Department of Pediatrics, Seinäjoki Central Hospital, Seinäjoki, Finland; 2 Center for Child Health Research, Tampere University and University Hospital, Tampere, Finland; 3 Department of Medical Microbiology and Immunology, University of Turku, Turku, Finland; 4 Department of Pediatrics and Adolescent Medicine, Turku University Hospital and Child and Youth Research Institute, University of Turku, Turku, Finland; 5 Department of Medical Microbiology, Capital Medical University, Beijing, China

## Abstract

Innate immunity receptors play a critical role in host defence, as well as in allergy and asthma. The aim of this exploratory study was to evaluate whether there are associations between *TLR7* rs179008, *TLR8* rs2407992, *TLR9* rs187084 or *TLR10* rs4129009 polymorphisms and viral findings, clinical characteristics or subsequent wheezing in infants with bronchiolitis. In all, 135 full-term infants were hospitalized for bronchiolitis at age less than 6 months: 129 of them were followed-up until the age of 1.5 years. The outcome measures were repeated wheezing, use of inhaled corticosteroids, atopic dermatitis during the first 1.5 years of life and total serum immunoglobulin E (IgE). There were no significant associations between the genotypes or allele frequencies of *TLR7* rs179008, *TLR8* rs2407992, *TLR9* rs187084 or *TLR10* rs4129009 polymorphisms and clinical characteristics or the severity of bronchiolitis during hospitalization. During follow-up, repeated wheezing was more common in children with *TLR9* rs187084 variant genotype CC (30.5%) than in children with *TLR9* wild-type genotype TT (12.2%) (p = 0.02, aOR 2.73, 95% CI 1.02–7.29). The *TLR10* rs4129009 minor allele G was associated with elevated total serum IgE. *TLR9* rs187084 gene polymorphism may be associated with post-bronchiolitis wheezing, and *TLR10* rs4129009 gene polymorphism may be associated with atopy.

Bronchiolitis is the most common lower respiratory tract infection (LRTI) in young children^1^. Among the various respiratory viruses causing bronchiolitis, respiratory syncytial virus (RSV) is the single most important one^2^. The clinical course of primary RSV infection is highly variable, and genetic variations in genes regulating the immune response evidently are important in determine whether a child suffers from a mild upper respiratory infection or a more severe LRTI- like bronchiolitis after exposure to RSV^3^.

Bronchiolitis in infancy is associated with subsequent wheezing in early childhood^4^. The mechanisms of virus-induced wheezing and the role of airway hyper-responsiveness, however, are poorly understood. Signalling via toll-like receptors (TLRs) seems to play an important role in triggering airway inflammation^5^,^6^.

TLRs are key molecules in innate immunity that detect conserved structures, which are present in a broad range of pathogens, and either promote or inhibit inflammatory and immune responses^7^. TLR3, TLR7, TLR8 and TLR9 recognize viral products responding to viral double-stranded (ds) RNA, viral single-stranded (ss) RNA or cytotoxic granule proteins containing DNA from certain bacteria and some viruses, respectively^8^,^9^. TLR10 is, in addition to TLR1, TLR2 and TLR6, a member of the TLR2 subfamily. Although TLR10 is a pattern-recognition protein without known ligand specificity, there is evidence that it is a modulatory receptor with mainly inhibitory properties^10^.

It has been reported that genetic factors might influence susceptibility to RSV infection in early life. *TLR4* gene polymorphisms have been associated with severe RSV infection^11^,^12^. In addition, *TLR9* and *TLR10* gene polymorphisms^13^ and *TLR 3* gene polymorphisms^14^ were associated with RSV bronchiolitis.

As the innate immune system is an important link between environmental exposure and the regulation of cytokine responses, alterations in innate immunity may be fundamental to the balance between Th1- and Th2-oriented responses and to the subsequent development of asthma and allergy. In a recent review on the relationship between genes encoding TLRs and asthma risk, an association was found between *TLR9* gene polymorphisms and asthma^15^. There is more evidence that polymorphisms in the *TLR7* and *TLR8* genes are related to asthma and allergy^16^,^17^,^18^, as well as to susceptibility to respiratory viral infections^19^,^20^.

We have prospectively followed-up a group of children hospitalized for bronchiolitis at age less than 6 months. We have earlier studied the *TLR1*rs5743618, *TLR2* rs5743708, *TLR3* rs3775291, *TLR4* rs4986790 and *TLR6* rs5743810 polymorphisms, and reported their associations with bronchiolitis and post-bronchiolitis outcomes. The aim of the present study was to complement this exploratory study series by evaluating whether the *TLR7* rs179008, *TLR8* rs2407992, *TLR9* rs187084 and *TLR10* rs4129009 polymorphisms are associated with the presence, clinical characteristics and viral etiology of bronchiolitis in early infancy. In addition, we studied whether there are associations between these polymorphisms and post-bronchiolitis outcomes including subsequent infections, post-bronchiolitis wheezing, need for corticosteroid treatment and atopic manifestations until the age of 1.5 years.

## Results

### Cases versus controls

The genotypes and allele frequencies of cases (children hospitalized with bronchiolitis) and controls (healthy children from a birth cohort study) did not differ substantially with respect to *TLR7* rs179008, *TLR8* rs2407992*, TLR9* rs187084 or *TLR10* rs4219009 polymorphisms (Table 1). Because *TLR7* rs179008 and *TLR8* rs2407992 are located on the X chromosome, the data are given separately for boys and girls. The studied alleles of *TLR9* and *TLR10* were in HWE. Because *TLR7* and *TLR8* genes are located on the X chromosome, HWE was not studied.

### Hospitalization in infancy

The mean age of the 135 bronchiolitis patients was 10.0 weeks (range 1–25 weeks, SD 6.94) during hospitalization. The study included 65 (48.1%) boys (Table 2). The causative virus was RSV in 101 (74.8%) cases and rhinovirus in 11 (8.1%) cases. Influenza A virus was found in 7 (5.2%) and human metapneumovirus in 2 (1.5%) cases, and parainfluenzavirus type 3 (PIV3) was found in 1 (0.8%) case. Two different viruses were detected in 8 (5.9%) samples: rhinovirus and adenovirus in two, and rhinovirus and bocavirus in two samples, and RSV and adenovirus, RSV and bocavirus, Influenza A and bocavirus, and PIV3 and adenovirus, each in one sample. There were no differences in the clinical picture or in the outcomes between cases with multiple viruses vs. a single virus detected. The sample was negative for studied viruses in 13 (9.6%) cases.

There were no significant associations between the *TLR7* rs179008 or *TLR8* rs4207992 genotypes or allele frequencies and the clinical characteristics of bronchiolitis in females (Table 3). Instead, the presence of the minor allele T in the *TLR7* rs179008 gene in males was associated with the RSV aetiology of bronchiolitis (100% *vs.* 70.4% in those with the major allele A) and with the need for feeding support (54.5% *vs.* 24.1%, respectively) (Table 4). The viral aetiology of bronchiolitis, and the need for feeding support or oxygen supplementation during bronchiolitis hospitalization did not differ between the children with different genotypes or allele frequencies of *TLR9* rs187084 or *TLR10* rs4219009 (Table 5). The LOS in hospital was roughly equal in all the groups constructed on the basis of *TLR7* rs179008, *TLR8* rs4207992, *TLR9* rs187084 or *TLR10* rs4219009 polymorphisms.

### Follow-up for 1.5 years

In all, 129 toddlers attended the follow-up visit at the mean age of 18.1 months (range 13–25 months, SD 2.35). There were 24 (19.4%) children who had presented with repeated wheezing (≥2 episodes) during the follow-up period and 16 (12.4%) children who had used inhaled corticosteroids (ICSs). Parents reported that 30 children (23.3%) had doctor-diagnosed food allergy and 18 children (14.0%) had doctor-diagnosed atopic dermatitis during the first 1.5 years of life (Table 2). In addition, 63 children (48.8%) had suffered from recurrent (≥3) otitis media. Total serum IgE was measured from 125 children: among them, the mean total serum IgE was 43.4 IU/ml (SD 95.9, range 1–621).

In males, the presence of the *TLR8* minor allele C was associated with recurrent otitis media, which was present in 22 (68.8%) of the 32 minor allele C carriers vs. in 13 of the 33 (39.4%) major allele G carriers (p = 0.04) (Table 6). However, the significance of this association was lost when adjusted for age (OR 2.75, 95% CI 0.96–7.85). In females, the result was of the same direction but not statistically significant (p = 0.06) (Table 7). There was a non-significant finding that the *TLR7* major allele A, when present as homozygous, was connected to recurrent otitis media in females (p = 0.07) (Table 7), but this was not seen in males (Table 6).

Former bronchiolitis patients with the homozygous variant *TLR9* rs187084 genotype CC had repeated wheezing more often (30.5%, p = 0.02) than children with the wild-type genotype TT (12.2%) during the follow-up period until the age of 1.5 years (Table 8). The OR was 2.66 (95% CI 1.01–7.06) when adjusted for age and sex, and 2.73 (95% CI 1.02–7.29) when adjusted further for atopic dermatitis. There were no significant associations between *TLR7* rs179008, *TLR8* rs2407992 or *TLR10* rs4219009 gene polymorphisms and repeated wheezing.

Children with *TLR10* rs4219009 genotype AG (no child had the GG genotype) had more often had elevated total serum IgE than children with *TLR10* genotype AA (Table 7). The OR was 3.79 (95%CI 1.25–11.56) when adjusted for age and sex. There were no significant associations between *TLR7* rs179008, *TLR8* rs2407992 or *TLR9* rs187084 gene polymorphisms and total serum IgE, food allergy or atopic dermatitis during the first 1.5 years of life.

## Discussion

Our study was an exploratory study on the possible association between the *TLR7* rs179008, *TLR8* rs4207992, *TLR9* rs187084 or *TLR10* rs4219009 gene polymorphisms and characteristics of bronchiolitis in early infancy or post-bronchiolitis outcomes. The main result was that the *TLR9* rs187084 polymorphism was associated with repeated wheezing until the age of 1.5 years after bronchiolitis at age less than 6 months. One-third of children who were homozygous for the variant genotype CC had suffered from repeated wheezing, compared to 12.2% of other former bronchiolitis patients. Another important result was that the *TLR10* rs4219009 minor allele G was associated with elevated total serum IgE in infancy. However, such associations were not seen in the case of clinical findings, i.e., food allergy or atopic dermatitis during the first 1.5 years of life. Both these results remained significant when adjusted for the most important confounders. In addition, we revealed a preliminary association between the presence of the *TLR8* rs4207992 minor allele C and recurrent otitis media in males but not in females.

However, polymorphisms *TLR7* rs179008, *TLR8* rs4207992, *TLR9* rs187084 and *TLR10* rs4219009 were not associated with the characteristics of bronchiolitis, including those reflecting bronchiolitis severity. All patients with bronchiolitis required hospitalization; one-fourth received supplementary oxygen; one-third required feeding support during hospitalization. Viruses such as RSV are recognized after invasion of the cells by those viral-sensing TLRs and other pattern-recognizing proteins, which are located within endosomes^21^.

In previous studies, *TLR4* gene polymorphisms have been associated with RSV infections^11^, and there is preliminary evidence that *TLR3* gene polymorphism^14^, and *TLR9* and *TLR10* gene polymorphisms as well^11^,^13^, may be associated with RSV bronchiolitis. In this exploratory study, bronchiolitis cases and population-based controls did not differ significantly for genotypes or allele frequencies of *TLR7* rs179008, *TLR8* rs4207992, *TLR9* rs187084 and *TLR10* rs4219009 polymorphisms.

In a recent systematic review and meta-analysis, *TLR9* rs5743836 gene polymorphism was associated with increased asthma risk^15^. TLR9 recognizes bacterial and viral CpG-DNA^22^. In an experimental study, the 1237 T/C single nucleotide polymorphisms (SNP) of TLR9 rs5743836 generated a functional IL-6 response element. Mononuclear cells carrying this SNP, when exposed to IL-6, responded by producing more TLR9, which then exacerbated the cellular response to the TLR9 ligand CpG^23^. IL-6 is a pro-inflammatory cytokine that may contribute to impaired lung function in allergic asthma^24^. In another study, the TLR9 rs5743836 variant genotype was associated with diminished lung function and chronic obstructive pulmonary disease (COPD)^25^. This effect was thought to be caused by alveolar macrophage dysfunction. However, there were no associations with TLR9 rs187084 SNPs^25^. Nonetheless, *TLR9* rs187084 polymorphism as applied in this study has also been shown to be functional^26^, being associated with immunity against certain infectious diseases such as tuberculosis and malaria^26^. Moreover, that polymorphism was associated with airway hyper-responsiveness in four population-based samples from Canada and Australia^27^. In our present study, the *TLR9* rs187084 polymorphism was associated with repeated post-bronchiolitis wheezing. In this same cohort, *TLR3* rs3775291 gene polymorphism was associated with repeated wheezing after bronchiolitis in infancy^14^.

The association of the *TLR10* gene with asthma has been documented in European-American populations^28^. Two-thirds of children who have repeated post-bronchiolitis wheezing until the age of two years are “transient wheezers” which means that they will grow out of their wheezing tendency by or at school age^29^. The risk of allergic asthma that is associated with the *TLR10* gene polymorphism^28^ was minor in our patients with bronchiolitis in early infancy. However, we found an association between *TLR10* rs4219009 gene polymorphism and elevated total serum IgE, and based on many studies, atopy and atopic sensitization are well-known risk factors of allergic asthma, in post-bronchiolitis cohorts as well^30^.

*TLR7* rs179008 and *TLR8* rs4207992 gene polymorphisms, located on the X chromosome, were not associated with post-bronchiolitis outcome measures in either boys or girls. In earlier studies, *TLR7* and *TLR8* polymorphisms were associated with asthma and allergic rhinitis^16^,^17^. Our finding is in disagreement with the results of a previous study^16^, in which the *TLR8* rs4207992 gene polymorphism, also used in the present study, was associated with asthma, atopic dermatitis, allergic rhinitis and elevated serum allergen-specific IgE. In experimental studies, concomitant *TLR7* gene deficiency and early *Pneumovirus* infection predisposed mice toward the development of asthma-like pathology^20^. In the present cohort, 19.4% of children suffered from repeated post-bronchiolitis virus-induced wheezing until 1.5 years of age. However, allergic asthma may develop later in childhood, even after many symptom-free years^29^.

There were certain limitations in the present study. The number of patients was relatively small for a genetic study. The small number of patients meant a risk of type-2 statistical errors. On the other hand, we carried out multiple analyses for polymorphisms of four different TLR encoding genes, which risked type-1 statistical errors. Because the data on *TLR7*, *TLR8*, *TLR9* and *TLR10* genes as risk factors for bronchiolitis, early-childhood wheezing and childhood asthma are mainly lacking, our study was an exploratory one with no purpose to test hypotheses, but rather to produce hypotheses for later confirmatory studies^31^,^32^. Therefore, we did not make any statistical multiplicity corrections. The main results were confirmed with multivariate tests adjusted for the most important confounding factors.

The strengths of the present study were the prospective design, an extensive virological test panel available during hospitalization for bronchiolitis and careful data collection during bronchiolitis and at the subsequent control visit including *e.g.* daily diaries recordings by parents. The homogeneity of study populations, as in the present study, is a clear benefit in genetic studies.

In conclusion, we found preliminary evidence that *TLR9* polymorphisms may be associated with post-bronchiolitis wheezing. This finding needs to be confirmed or ruled out in subsequent confirmatory studies.

## Methods

### Bronchiolitis patients

Previously healthy, full-term infants hospitalized for bronchiolitis at less than 6 months of age during the period December 1, 2001 through May 31, 2002 and during the period from October 28, 2002 through May 31, 2004 were enrolled in the study. In all, 187 eligible infants were hospitalized for the first time during the two study periods. Appropriate clinical data including viral findings and gene polymorphisms were available from 135 infants^33^. Bronchiolitis was defined as an acute LRTI characterized by rhinorrhea, cough, and diffuse wheezes and/or crackles^1^. The viral aetiology of bronchiolitis was studied by antigen detection and polymerase chain reaction (PCR) in nasopharyngeal aspirates (NPA), as described recently^33^. The studied viruses were RSV, human rhinovirus (hRV), human metapneumovirus (hMPV), InfluenzaA virus, Parainfluenza3 virus (PIV3), adenovirus and human bocavirus (hBoV). Data on disease severity, such as the need for supplementary oxygen and feeding support, and the length of hospital stay (LOS) were recorded during the inpatient care^33^. Supplementary oxygen was given when oxygen saturation (SaO_2_) measured with pulse-oximetry was less than 94%, and feeding support was defined as a need for intravenous fluids or feeding via a nasogastric tube.

After hospitalization for bronchiolitis, the children were invited to a follow-up visit at 1.5 years of age (on average), and 129 (92.8% of those invited; 69.0% of those hospitalized) attended. At the follow-up visit, the parents were interviewed using a structured questionnaire on the occurrence of otitis media, and wheezing episodes, the prescription of antibiotics, and the use of corticosteroids for wheezing after hospitalization for bronchiolitis^34^. The parents had recorded a diary during the 1.5 years post-bronchiolitis follow-up period, including descriptions of all infections and wheezing periods verified by a family doctor or a paediatrician. The study physician and the parents checked together the diaries at the control visit. The doctor-diagnosed episodes were recorded for the analyses.

Food allergy and atopic dermatitis, although reported by parents, were registered during hospitalization and at the control visit on average 1.5 years later—only doctor-diagnosed cases were included. A blood sample for immunoglobulin E (IgE) measurement was taken at the follow-up visit at the age of 1.5 years. Total serum IgE was determined by electro-chemiluminescence (ECLIA, Roche Diagnostics GmbH, Mannheim, Germany) according to laboratory practice. Serum IgE was considered to be elevated if the concentration was more than +2SD above the mean of non-atopic Finnish children (>60 IU/ml)^35^. Corticosteroid treatment meant maintenance medication periods with inhaled corticosteroids (ICSs) for repeated wheezing or suspected asthma. Repeated wheezing was defined as two or more wheezing episodes during the post-bronchiolitis follow-up period.

Frozen whole-blood samples were available for *TLR7* rs179008, *TLR8* rs2407992, and *TLR10* rs4129009 genotyping from all 129 children. Samples for *TLR9* rs187084 genotyping were available from 127 children.

### Genetic studies

The genotyping of *TLR8* rs2407992 (2040 C/G) was performed by pyrosequencing as described for *TLR3* rs3775291 (1234 C/T)^14^. For *TLR7* rs179008 (171 A/T), the PCR products were first purified using the QIA quick PCR Purification Kit (Qiagen, Hilden, Germany) according to the manufacturer’s protocol. PCR products with low deoxyribonucleic acid (DNA) content were eluted to 30 μl of elution buffer. After purification, the PCR products were pipetted to a 96-well plate (5 μl) together with *TLR7* rs179008 (171 A/T) forward primer (1.6 μl). The 96-well plate was sent to the Institute for Molecular Medicine laboratory in Helsinki, Finland for sequencing. *TLR9* rs187084 (1486 T/C) genotyping was performed using BspTI restriction enzyme (Thermo Fisher Scientific, Waltham, USA) for the digestion of PCR product^36^. High-resolution melting analysis (HMR) (Roche Diagnostics Light Cycler 480, Basel, Switzerland), was used to genotype *TLR10* rs4129009 (2322 A/G) as described earlier^36^. The methods were described recently in more details^36^. The PCR and sequencing primers used for the *TLR7*, *TLR8, TLR9*, *and TLR10* genes are described in Table 9. All the primers were purchased from Sigma-Aldrich, Finland.

### Controls

*TLR7* rs179008 and *TLR8* rs2407992 polymorphisms were examined in two-month-old healthy Finnish infants, who participated in the Steps to Children’s Healthy Development and Wellbeing study (STEPS), which is a prospective birth cohort study of approximately 1,800 children^37^. No selection criteria were applied in the recruitment of children for the STEPS Study or for inclusion in the subgroup with TLR genotyping. DNA was extracted from blood collected at the age of two to three months in 412 subjects, who visited the study clinic in Turku, Finland^37^. Only 328 children were included as controls in the present study for *TLR7*rs 179008*, TLR8* rs2407992 and *TLR10* rs 4129009 polymorphisms, due to the limited amount of DNA available from the infants who were not included. For *TLR 9* rs187084, there was enough DNA to analyze only 270 samples. The genotyping processes were identical in the bronchiolitis cohort and in the controls, except for the *TLR10* rs4129009 gene. Determination of that polymorphism was performed by digestion similarly to *TLR9* rs187084 in the control group, and by HRMA in the bronchiolitis cohort.

### Ethics

The study was carried out in accordance with the approved guidelines of the WMA Declaration of Helsinki. Before we enrolled the children we obtained informed parental consent, including the use of samples for genetic studies on bronchiolitis and asthma risk, both during hospitalization and at the control visit. The protocol of the study was approved by the Ethics Committee of the Tampere University Hospital District, Tampere, Finland. The personal data of the study subjects were not given to the two laboratories that performed the genetic studies, the National Institute of Health and Welfare, Turku, Finland, and the Institute for Molecular Medicine, Helsinki, Finland. The genotyping of control children was done within the STEPS study, which was carried out in accordance with the approved guidelines of the WMA Declaration of Helsinki. The protocol was approved by the Ethics Committee of the Hospital District of Southwest Finland, Turku, Finland. The parents of participating children provided their written, informed consent.

### Statistical analyses

Data were analyzed using SPSS package version 23.0 (IBM Corp. NY, USA). Chi square and Fisher’s exact tests were used for categorized variables. Student’s t-test was used for normally distributed variables. The Mann–Whitney test was used for non-normally distributed continuous variables. The results were expressed as frequencies, proportional frequencies, means, medians and standard deviations (SD).

Logistic regression was used for the multivariate analyses, adjusted for age, sex and atopic dermatitis during the first 1.5 years of life when appropriate. The results were expressed as odds ratios (OR) and 95% confidence intervals (95% CI). The FINETTI program was used to evaluate the Hardy–Weinberg equilibrium (HWE) of the studied *TLR9* and *TLR10* alleles studied.

## Additional Information

**How to cite this article**: Nuolivirta, K. *et al.* Post-bronchiolitis wheezing is associated with toll-like receptor 9 rs187084 gene polymorphism. *Sci. Rep.* **6**, 31165; doi: 10.1038/srep31165 (2016).

## Figures and Tables

**Table 1:** The genotypes and allele frequencies of *TLR7* rs179008 (171 A/T), *TLR8* rs2407992 (2040 C/G), *TLR9* rs187084 (1486 T/C) and *TLR10* rs4219009 (2322 A/G) in the bronchiolitis patients (cases) and population-based controls.

Genotypes and allele frequencies	Cases, No. (%)	Controls, No. (%)
*TLR7* females AA	43/70 (61.4)	77/145 (53.1)
*TLR7* females AT	23/70 (32.9)	53/145 (36.6)
*TLR7* females TT	4/70 (5.7)	15/145 (10.3)
*TLR7* females, major allele A	109/140 (77.9)	207/290 (71.4)
*TLR7* females, minor allele T	31/140 (22.1)	83/290 (28.6)
*TLR7* males, allele A present	54/65 (83.1)	116/168 (69.0)
*TLR7* males, allele T present	11/65 (16.9)	52/168 (31.0)
*TLR8* females GG	19/70 (27.1)	43/144 (29.8)
*TLR8* females GC	41/70 (58.6)	70/144 (48.6)
*TLR8* females CC	10/70 (14.3)	31/144 (21.6)
*TLR8* females, major allele G	79/140 (56.4)	156/288 (54.2)
*TLR8* females, minor allele C	61/140 (43.6)	132/288 (45.8)
*TLR8* males, allele G present	32/65 (49.2)	89/168 (53.0)
*TLR8* males, allele C present	33/65 (50.8)	79/168 (47.0)
*TLR9* TT	42/133 (31.6)	87/270 (32.0)
*TLR9* TC	55/133 (41.4)	130/270 (48.0)
*TLR9* CC	36/133 (27.1)	53/270 (20.0)
*TLR9* major allele T	139/266 (52.3)	304/540 (56.3)
*TLR9* minor allele C	127/266 (47.7)	236/540 (43.7)
*TLR10* AA	113/135(83.7)	271/328 (82.6)
*TLR10* AG	21/135 (15.6)	47/328 (14.4)
*TLR10* GG	1/135 (0.7)	10/328 (3.0)
*TLR10* major allele A	247/270 (91.5)	589/656 (89.8)
*TLR10* minor allele *G*	23/270 (8.5)	67/656 (10.2)

The test showed heterozygosis of the *TLR7* gene in four controls and heterozygosis of the *TLR8* gene in four controls, although the subjects were male. In one male control, the test results showed heterozygosis of both *TLR7* and *TLR8*. These seven controls were deleted from the analyses. There were no statistically significant differences between cases and controls.

**Table 2:** Clinical characteristics of the bronchiolitis population during hospitalization and follow-up visit at the mean age of 1.5 years.

Infants during bronchiolitis N = 135	
Age (mean, range, SD)	10.0 weeks (1–25, SD 6.94)
Boys N(%)	65 (48.1)
RSV N (%)	101 (74.8)
Feeding support N (%)	45 (33.3)
Oxygen supplementation N(%)	25 (18.5)
Length of hospital stay (mean, range, SD)	4.74 days (0–22, Sd 4.74)
**Children at follow-up visit N = 129**	
Age (mean, range, SD)	18.1 months (13–25, SD 2.35)
Boys	62 (48.1)
Post-bronchiolitis wheezing N (%)	24 (19.4)
Inhaled corticosteroids	16 (12.4)
Food allergy	30 (23.3)
Atopic dermatitis	18 (14.0)

**Table 3:** Genotypes and major allele frequencies of *TLR7* rs179008 and *TLR8* rs2407992 genes in 70 female patients in relation to clinical characteristics of bronchiolitis.

TLR7 females N = 70	AA N = 43, No. (%)	AT N = 23, No. (%)	TT N = 4, No. (%)	Major allele AN = 109, No. (%)
Virus RSV N = 52	34 (79.1) p = 0.19	15 (65.2)	3 (75.0) p = 0.73	83 (76.1) p = 0.71
Oxygen required N = 16	9 (20.9) p = 0.42	6 (26.1)	1 (25.0) p = 0.67	24 (22.0) p = 0.73
Feeding support N = 28	19 (67.9) p = 0.26	7 (30.4)	2 (50.0) p = 0.53	45 (41.3) p = 0.70
LOS, mean in days (standard deviation)	5.4 (SD 3.7) p = 0.51	5.0 (SD 3.0)	4.0 (SD 1.8) p = 0.48	
**TLR8 females N = 70**	**GG N = 19, No. (%)**	**GC N = 41, No. (%)**	**CC N = 10, No. (%)**	**Major allele G N = 79, No. (%)**
Virus RSV N = 52	14 (73.7) p = 0.59	30 (73.2)	8 (80.0) p = 0.50	58 (73.4) p = 0.92
Oxygen required N = 16	5 (26.3) p = 0.45	10 (24.4)	1 (10.0) p = 0.28	20 (25.3) p = 0.56
Feeding support N = 28	9 (47.4) p = 0.31	15 (36.4)	4 (40.0) p = 0.64	33 (41.8) p = 0.75
LOS, mean in days (standard deviation)	4.9 (SD 2.1) p = 0.69	5.4 (SD 3.9)	4.8 (SD 3.4) p = 0.72	

The length of hospital stay (LOS) was mean 5.2 days (SD 3.4, range 1–25) in all 70 cases. Major allele vs minor allele.

**Table 4:** Genotypes and major allele frequencies of *TLR7* rs179008 and *TLR8* rs2407992 genes in 65 male patients in relation to the clinical characteristics of bronchiolitis.

TLR7 males N = 65	A present N = 54, No. (%)	T present N = 11, No. (%)
Virus RSV N = 49	38 (70.4) **p = 0.03**	11 (100)
Oxygen required N = 9	6 (11.1) p = 0.71	3 (27.3)
Feeding support N = 19	13 (24.1) **p = 0.05**	6 (54.5)
LOS, mean in days (standard deviation)	4.0 (SD 2.7) p = 0.06	5.8 (SD 3.7)
**TLR8 males N = 65**	**G present N = 32 (%)**	**C present N = 33 (%)**
Virus RSV N = 49	26 (81.3) p = 0.21	23 (69.7)
Oxygen required N = 9	6 (18.8) p = 0.22	3 (9.1)
Feeding support N = 19	9 (28.1) p = 0.53	10 (30.3)
LOS, mean in days (standard deviation)	4.1 (SD 3.2) p = 0.69	4.4 (SD 2.7)

The length of hospital stay (LOS) was mean 4.3 days (SD 3.0, range 0–15) in all 65 cases.

**Table 5:** Genotypes and major allele frequencies of *TLR9* rs187084 (N = 133) and *TLR10* rs4219009 (N = 135) genes in bronchiolitis patients in relation to the clinical characteristics of bronchiolitis.

TLR9 N = 133	TT N = 42, No. (%)	CT N = 55, No. (%)	CC N = 36, No. (%)	Major allele T N = 139, No. (%)
Virus RSV N = 99	33 (78.6) p = 0.78	39 (70.9)	27 (75.0) p = 0.97	105 (75.5) p = 0.87
Oxygen required N = 25	7 (16.7) p = 0.43	13 (23.6)	5 (13.9) p = 0.27	27 (19.4) p = 0.82
Feeding support N = 45	14 (33.3) p = 0.55	21 (38.9)	10 (27.8) p = 0.25	49 (35.3) p = 0.72
LOS, mean in days (standard deviation)	5.10 (SD 3.80) p = 0.85	4.45 (SD 2.41)	4.61 (SD 3.58) p = 0.34	
**TLR10 N = 135**	**AA N = 113, No. (%)**	**AG N = 21, No. (%)**	**GG N = 1,**	**Major allele A N = 247, No. (%)**
Virus RSV N = 101	84 (74.3) p = 0.50	16 (76.2)	1	184 (74.5) p = 0.
Oxygen required N = 25	22 (19.5) p = 0.38	2 (9.5)	1	46 (18.6) p = 0.
Feeding support N = 47	40 (35.4) p = 0.48	6 (28.6)	1	86 (34.8) p = 0.
LOS, mean in days (standard deviation)	4.74 SD 3.25 p = 0.94	4.52 SD 3.04	NC	

The length of hospital stay (LOS) was mean 4.7 days (SD 3.2, range 0–22) in all 133 cases.

NC not calculated. Major allele vs minor allele.

**Table 6:** Genotypes and major allele frequencies of *TLR7* rs179008 and *TLR8* rs2407992 genes in 62 male bronchiolitis patients in relation to outcome variables during the post-bronchiolitis follow-up until the age of 1.5 years.

TLR7 males Outcome variables, N = 62	A present N = 54, No. (%)	T present N = 11, No. (%)
Repeated (≥2 episodes) wheezing N = 15	14 (27.4) p = 0.19	1 (9.1)
Inhaled corticosteroids N = 11	10 (19.6) p = 0.37	1 (9.1)
Atopic dermatitis N = 10	10 (19.6) p = 0.12	0
Food allergy N = 15	11 (21.6) p = 0.25	4 (36.4)
IgE ≥ 60 N = 10/59	8 (15.7) p = 0.60	2 (18.2)
Recurrrent (≥3) otitis media N = 35	26 (50.9) p = 0.06	9 (81.8)
**TLR8 males Outcome variables, N = 62**	**G present N = 30, No. (%)**	**C present N = 32, No. (%)**
Repeated (≥2 episodes) wheezing N = 15	5 (16.7) p = 0.15	10 (31.3)
Inhaled corticosteroids N = 11	3 (10.0) p = 0.11	8 (25.0)
Atopic dermatitis N = 10	2 (6.7) **p = 0.05**	8 (25.0)
Food allergy N = 15	7 (23.3) p = 0.56	8 (25.0)
IgE ≥ 60 N = 10/59	3 (10.0) p = 0.19	7 (21.9)
Recurrrent (≥3) otitis media N = 35	13 (39.4) **p = 0.04**	22 (68.8)

**Table 7:** Genotypes and major allele frequencies of *TLR7* rs179008 and *TLR8* rs2407992 genes in 67 female bronchiolitis patients in relation to outcome variables during the post-bronchiolitis follow-up until the age of 1.5 years.

TLR7 females Outcome variable, N = 67	AA N = 42, No. (%)	AT N = 21, No. (%)	TT N = 4, No. (%)	Major allele A N = 105, No. (%)
Repeated (≥2 episodes) wheezing N = 9	4 (9.5) p = 0.20	5 (23.8)	0 p = 0.55	13 (12.4) p = 1.0
Inhaled corticosteroids N = 5	2 (4.8) p = 0.27	3 (14.3)	0 p = 0.73	7 (6.7) p = 0.69
Atopic dermatitis N = 8	4 (9.5) p = 0.34	2 (9.5)	2 (50.0) p = 0.07	10 (9.5) p = 0.16
Food allergy N = 15	12 (28.6) p = 0.10	3 (14.3)	0 p = 0.35	27 (25.7) p = 0.20
IgE ≥ 60 N = 10/65	4 (9.5) p = 0.12	6 (28.6)	0 p = 0.60	14 (14.3) p = 0.41
Recurrrent (≥3) otitis media N = 28	21 (50.0) p = 0.07	6 (28.6)	1 (25.0) p = 0.44	48 (46.7) p = 0.24
**TLR8 females Outcome variable, N = 67**	**GG N = 18, No. (%)**	**GC N = 39, No. (%)**	**CC N = 10, No. (%)**	**Major allele G N = 75, No. (%)**
Repeated (≥2 episodes) wheezing N = 9	2 (11.1) p = 0.55	7 (17.9)	0 p = 0.21	10 (13.3) p = 0.62
Inhaled corticosteroids N = 5	2 (11.1) p = 0.41	3 (7.7)	0 p = 0.43	7 (9.3) p = 0.52
Atopic dermatitis N = 8	2 (11.1) p = 0.63	6 (15.4)	0 p = 0.25	10 (13.3) p = 0.62
Food allergy N = 15	3 (16.7) p = 0.37	11 (28.2)	1 (10.0) p = 0.29	17 (22.7) p = 0.94
IgE ≥ 60 N = 10/65	3 (16.7) p = 0.57	7 (17.9)	0 p = 0.20	13 (17.3) p = 0.45
Recurrrent (≥3) otitis media N = 28	8 (44.4) p = 0.50	14 (35.9)	6 (60.0) p = 0.18	30 (40.0) p = 0.76

Major allele vs minor allele.

**Table 8:** Genotypes and major allele frequencies of *TLR9* rs187084 (N = 127) and *TLR10* rs4219009 (N = 129) genes in bronchiolitis patients in relation to outcome variables during the post-bronchiolitis follow-up until the age of 1.5 years.

TLR9 Outcome variables, N = 127	TT N = 41, No. (%)	TC N = 53, No. (%)	CC N = 33, No.(%)	Major allele T N = 135, No.(%)
Repeated (≥2 episodes) wheezing N = 24	5 (12.2) p = 0.14	8 (15.1)	11 (30.5) **p = 0.02**	18 (13.3) **p = 0.047**
Inhaled corticosteroids N = 16	3 (7.3) p = 0.17	7 (13.2)	6 (18.2) p = 0.20	13 (9.6) p = 0.18
Atopic dermatitis N = 18	9 (22.0) p = 0.07	4 (7.5)	5 (15.2) p = 0.53	22 (16.3) p = 0.37
Food allergy N = 30	7 (17.1) p = 0.17	14 (26.4)	9 (27.3) p = 0.36	28 (20.7) p = 0.37
IgE ≥ 60 N = 20/123	7 (17.1) p = 0.46	7 (13.2)	6 (18.2) p = 0.46	21 (15.6) p = 0.94
Recurrrent (≥3) otitis media N = 62	22 (53.7) p = 0.29	21 (39.6)	19 (57.6) p = 0.17	65 (48.1) p = 0.89
**TLR10 Outcome variables, N = 129**	**AA N = 109, No. (%)**	**AG N = 19, No. (%)**	**GG N = 1**	**Major allele A N = 237, No. (%)**
Repeated (≥2 episodes) wheezing N = 24	20 (18.3) p = 0.54	4 (21.1)	0	44 (18.6) p = 0.78
Inhaled corticosteroids N = 16	13 (11.9) p = 0.47	3 (15.8)	0	29 (12.2) p = 0.73
Atopic dermatitis N = 18	16 (14.7) p = 0.44	2 (10.5)	0	34 (14.3) p = 1.0
Food allergy N = 30	27 (24.8) p = 0.26	3 (15.8)	0	57 (24.1) p = 0.59
IgE ≥ 60 N = 20/124	13(11.9) p = 0.015	7 (36.8)	0	33 (13.9) p = 0.08
Recurrrent (≥3) otitis media N = 63	54 (49.5) p = 0.45	9 (47.4)	0	117 (49.4) p = 0.84

Major allele vs. minor allele.

**Table 9:** Primer sequences, allele and amino acid changes in toll-like receptor (TLR) genes TLR7 rs179008 (171A/T), TLR8 rs2407992 (2040C/G), TLR9 rs187084 (1486T/C) and TLR10 rs4129009 (2322A/G).

TLR7 11Gln > Leu (171A/T) (rs179008)
for 5′-AGATGTCTGGTATGTGGTT-3′rev 5′-TGATTCTTGGTATGTTTTAGA-3′
TLR8 651Leu > Leu (2040C/G) (rs2407992)
for 5′-TGGAAAGCAAGTCCCTGGTA-3′rev 5′-Biotin-AGTGAGACTCGCTGGCAAAT-3′seq 5′-ATCCCTTAATAGGCT-3′
TLR9 (silent mutation) (1486T/C) (rs187084)
for 5′-ACTATGGAGCCTGCCTGCCATGATACC-3′rev 5′-ATCCAGCCTTCTTACAAACCTCCCACC-3′restriction enzyme BspTI
TLR10 775Ile > Val (2322A/G) (rs4129009)
for 5′-CTTACTGGAACCCATTCCATTCTATTGC-3′rev 5′-TCAATGTACATCCCAACAGTGTATGTGG-3′restriction enzyme VspI
TLR10 775Ile > Val (2322A/G) (rs4129009)
for 5′-AGTTCATACATTTCTCTGGTGGCT-3′rev 5′-GTGGGCTTTTCTGGGCAAAC-3′

TLR7 SNP was detected by common sequencing, TLR8 SNP by pyrosequencing, TLR9 by digestion and TLR10 by high-resolution analysis.

## References

[b1] AAP. American Academy of Pediatrics. & Subcommittee on Diagnosis and Management of Bronchiolitis. Diagnosis and management of bronchiolitis. Pediatrics 118, 1774–1793 (2006). 17015575 10.1542/peds.2006-2223

[b2] Stockman L. J., Curns A. T., Anderson L. J. & Fisher-Langley G. Respiratory syncytial virus-associated hospitalizations among infants and young children in the United States, 1997–2006. Pediatr Infect Dis J 31, 5–9 (2012). 21817948 10.1097/INF.0b013e31822e68e6

[b3] Miyairi I. & DeVincenzo J. P. Human genetic factors and respiratory syncytial virus disease severity. Clin Microbiol Rev 21, 686–703 (2008). 18854487 10.1128/CMR.00017-08 PMC2570150

[b4] Piippo-Savolainen E. & Korppi M. Wheezy babies-wheezy adults? Review on long-term outcome until adulthood after early childhood wheezing. Acta Paediatr 97, 5–11 (2008). 18052998 10.1111/j.1651-2227.2007.00558.x

[b5] Duechs M. J. *et al.* TLR agonist mediated suppression of allergic responses is associated with increased innate inflammmation in the airways. Pulm Pharmacol& Ther 24, 203–214 (2011). 21195789 10.1016/j.pupt.2010.12.009

[b6] Chen K. *et al.* The active contribution of Toll-like receptors to allergic airway inflammation. Int Immunopharmacol 11, 1391–1398 (2011). 21624504 10.1016/j.intimp.2011.05.003 PMC7398422

[b7] Akira S. & Takeda K. Toll-like receptor signalling. Nat Rev Immunol 4, 499–511 (2004). 15229469 10.1038/nri1391

[b8] Iwasaki A. & Medzhitov R. Toll-like recptor control of the adaptive immune responses. Nat Immunol 5, 987–995 (2004). 15454922 10.1038/ni1112

[b9] Abreu M. T. & Arditi M. Innate immunity and Toll-like receptors:clinical implications of basic science research. J Pediatr 144, 241–249 (2004). 10.1016/j.jpeds.2004.01.057 15069387

[b10] Oosting M. *et al.* Human TLR10 is an anti-inflammatory pattern-recognition receptor. Proc Natl Acad Sci USA 111, E4478–E4484 (2014). 25288745 10.1073/pnas.1410293111 PMC4210319

[b11] Choi E. H., Lee H. J. & Chanock S. J. Human genetics and respiratory syncytial virus disease: current findings and future approaches. Curr Top Microbiol Immunol 372, 121–137 (2013). 24362687 10.1007/978-3-642-38919-1_6

[b12] Zhou J. *et al.* Genetic association of TLR4 Asp299Gly, TLR4 Thr399Ile, and CD14 C-159T polymorphisms with the risk of severe RSV infection: a meta-analysis. Influenza and Other Respiratory Viruses 10, 224–233 (2016). 26901241 10.1111/irv.12378 PMC4814857

[b13] Mailaparambil B., Krueger M., Heinze J., Forster J. & Heinzmann A. Polymorphisms of toll like receptors in the genetics of severe RSV associated disease. Dis Markers 25, 59–65 (2008). 18776592 10.1155/2008/619595 PMC3827794

[b14] Nuolivirta K. *et al.* Toll-like Receptor 3 L412F Polymorphisms in Infants With Bronchiolitis and Postbronchiolitis Wheezing. Pediatr. Infect. Dis. J. 31, 920–923 (2012). 22549436 10.1097/INF.0b013e31825aff25

[b15] Tizaoui K., Kaabachi W., Hamzaoui K. & Hamzaoui A. Association of single nucleotide polymorphisms in toll-like receptor genes with asthma risk: a systematic review and meta-analysis. Allergy Asthma Immunol Res 7, 130–140 (2015). 25729620 10.4168/aair.2015.7.2.130 PMC4341334

[b16] Moller-Larsen S. *et al.* Association analysis identifies TLR7 and TLR8 as novel risk genes in asthma and related disorders. Thorax 63, 1064–1069 (2008). 18682521 10.1136/thx.2007.094128

[b17] Nilsson D. *et al.* Toll-like receptor gene polymorphisms are associated with allergic rhinitis: A case control study. BMC Medic Genet 13, 66 (2012). 10.1186/1471-2350-13-66 PMC3459792 22857391

[b18] Pritchard A. L. *et al.* Asthma is associated with multiple alterations in anti- viral innate signalling pathways. PloS ONE 9, e106501 (2014). 25203745 10.1371/journal.pone.0106501 PMC4159236

[b19] Drake M. G. *et al.* Toll-like receptor 7 rapidly relaxes human airways. Am J Respir Crit Care Med 188, 664–672 (2013). 23924358 10.1164/rccm.201303-0442OC PMC3826186

[b20] Kaiko G. E. *et al.* Toll-like receptor 7 gene deficiency and early-life pneumovirus infection interact to predispose toward the development of asthma-like pathology in mice. J Allergy Clin Immunol 131, 1331–1339 (2013). 23561801 10.1016/j.jaci.2013.02.041

[b21] Pichlmair A. & Reis e Sousa C. Innate recognition of viruses. Immunity 27, 370–383 (2007). 17892846 10.1016/j.immuni.2007.08.012

[b22] Bezemer G. *et al.* Dual role of Toll-like receptors in asthma and chronic obstructive pulmonary disease. Pharmacol Rev 64, 337–358 (2012). 22407613 10.1124/pr.111.004622

[b23] Carvalho A. *et al.* The C allele of rs5743836 polymorphism in the human TLR9 promoter links IL-6 and TLR9 up-regulation and confers increased B-Cell proliferation. PloS ONE 6, e28256 (2011). 22132241 10.1371/journal.pone.0028256 PMC3223238

[b24] Neveu W. A. *et al.* Elevation of IL-6 in the allergic asthmatic airway is independent of inflammation but associates with loss of central airway function. Respir Res 11, 28 (2010). 20205953 10.1186/1465-9921-11-28 PMC2842243

[b25] Berenson C. S., Kruzel R. L., Wrona C. T., Mammen M. J. & Sethi S. Impaired innate COPD alveolar macrophage responses and Toll-like receptor-9 polymorphisms. PloS ONE 10, e.0134209 (2015). 10.1371/journal.pone.0134209 PMC4567310 26361369

[b26] Bharti D. *et al.* The role of TLR9 polymorphism in susceptibility to pulmonary tuberculosis. Immunogenetics 66, 675–681 (2014). 25248338 10.1007/s00251-014-0806-1

[b27] Daley D. *et al.* Analyses of associations with asthma in four asthma population samples from Canada and Australia. Hum Genet 125, 445–459 (2009). 19247692 10.1007/s00439-009-0643-8

[b28] Lazarus R. *et al.* TOLL-like receptor 10 genetic variation is associated with asthma in two independent samples. Am J Respir Crit Care Med 170, 594–600 (2004). 15201134 10.1164/rccm.200404-491OC

[b29] Brand P. L. *et al.* Definition, assessment and treatment of wheezing disorders in preschool children: an evidence-based approach. Eur Respir J 32, 1096–1110 (2008). 18827155 10.1183/09031936.00002108

[b30] Kotaniemi-Syrjänen A., Reijonen T. M., Korhonen K. & Korppi M. Wheezing requiring hospitalization in early childhood: predictive factors for asthma in a six-year follow-up. Pediatr Allergy Immunol 13, 418–425 (2002). 12485317 10.1034/j.1399-3038.2002.02091.x

[b31] Bender R. & Lange S. Adjusting for multiple testing—when and how? J Clin Epidemiol 54, 343–349 (2001). 11297884 10.1016/s0895-4356(00)00314-0

[b32] Streiner D. L. Best (but oft-forgotten) practices: the multiple problems of multiplicity-whether and how to correct for many statistical tests. Am J Clin Nutr doi: 10.3945/ajcn.115.113548 (2015). 26245806

[b33] Helminen M. *et al.* IL-10 gene polymorphism at −1082 A/G is associated with severe rhinovirus bronchiolitis in infants. Pediatr Pulmonol 43, 391–395 (2008). 18286551 10.1002/ppul.20793

[b34] Nuolivirta K. *et al.* Gene polymorphism of *IFNG* +874 T/A and *TLR4* +896 A/G and recurrent infections and wheezing in toddlers with history of bronchiolitis. Ped Infect Dis J 28, 1121–1123 (2009). 10.1097/INF.0b013e3181af37ee 19773677

[b35] Saarinen U. M., Juntunen K., Kajosaari M. & Björksten F. Serum Immunoglobulin E in atopic and non-atopic children aged 6 months to 5 years. A follow-up study. Acta Paediatr Scand 71, 489–494 (1982). 7136662 10.1111/j.1651-2227.1982.tb09457.x

[b36] Lauhkonen E. *et al.* Gene polymorphism of toll-like receptors and lung function at five to seven years of age after infant bronchiolitis. PloS ONE 11, e0146526 (2016). 26741133 10.1371/journal.pone.0146526 PMC4704821

[b37] Lagström H. *et al.* Cohort profile: Steps to the healthy development and well-being of children (the STEPS study). Int J Epidemiol 42, 1273–1284 (2013). 23143610 10.1093/ije/dys150

